# No Obesity Paradox—BMI Incapable of Adequately Capturing the Relation of Obesity with All-Cause Mortality: An Inception Diabetes Cohort Study

**DOI:** 10.1155/2014/282089

**Published:** 2014-08-07

**Authors:** Mohammadreza Bozorgmanesh, Banafsheh Arshi, Farhad Sheikholeslami, Fereidoun Azizi, Farzad Hadaegh

**Affiliations:** ^1^Prevention of Metabolic Disorders Research Center, Research Institute for Endocrine Sciences (RIES), Shahid Beheshti University of Medical Sciences, Tehran 1985717413, Iran; ^2^Endocrine Research Center, Research Institute for Endocrine Sciences (RIES), Shahid Beheshti University of Medical Sciences, Tehran 1985717413, Iran

## Abstract

*Background*. To reconcile “the obesity paradox,” we tested if (1) the contribution of anthropometric measures to mortality was nonlinear and (2) the confounding of hip circumference contributed to the obesity paradox recently observed among diabetic patients. *Methods*. We analyzed data of diabetic patients attending a community-based prospective, “Tehran lipid and glucose study.” In the mortality analysis, anthropometric measures—body mass index (BMI), waist, and hip circumference—were assessed using Cox models incorporating cubic spline functions. *Results*. During 12 990 person-years follow-up, BMI levels below 27 and those above 40 kg*·*m^−2^ were associated with increased mortality. When we added waist circumference to the BMI in the multivariate-adjusted model, the steepness of BMI-mortality association curve slope for values below 27 kg*·*m^−2^ increased, whereas the steepness of BMI-mortality association curve slope for values above this threshold decreased. Further adjusting the model for hip circumference, the steepness of the slopes of the association curve moved towards null on both extremes and no associations between BMI and all-cause mortality remained. *Conclusion*. BMI harbors intermixed positive and negative confounding effects on mortality of waist and hip circumference. Failing to control for the confounding effect of hip circumference may stymie unbiased hazard estimation and render conclusions paradoxical.

## 1. Background

The so-called “obesity paradox” has been observed in a variety of settings [1–5]. The U-shaped relationship of body mass index (BMI) with mortality has been demonstrated in general populations, yet, the definite excess mortality below 25 kg*·*m^−2^ has not been fully explained [6]. Researchers have defined a condition generally referred to as the metabolically obese normal weight phenotype. This phenotype is ascertained among normal weight individuals based on their BMI but present metabolic aspects of obesity. The condition has been observed to be increasingly prevailing over time [3, 7–9].

Along with the emergence of the concept of metabolically obese normal weight, the “obesity paradox” phenomenon came into notion [10, 11]. Obesity was long known to contribute to mortality [2]. Examining nonlinear contribution of the anthropometric indices of obesity, however, unveiled a U-shaped association between obesity measures and mortality, particularly among patients with chronic disease like hypertension [12], kidney disease [13], and heart failure [14].

No other chronic disease is as known to be related to obesity as diabetes. The role of obesity in diabetes was so ingrained that some investigators suggested the term “*DIABESITY* (*DIABE*tes + obe*SITY*)” for both conditions [15]. However, what hinders the term to become extensively used are findings indicating that diabetic patients who lost weight during follow-up or were of normal weight at the baseline had a higher mortality than diabetic patients who were overweight or obese [16, 17]. Participants of these studies had diabetes of unknown duration [18]. This makes it difficult to distinguish between the role of weight loss as an innocent bystander reflecting poorly controlled diabetes or as a mediator effectuating the impact of diabetes on death or as an independent risk factor [19]. To deal with this limitation, Carnethon et al. have conducted a study on a sample of participant with newly diagnosed diabetes to examine all-cause, cardiovascular, and noncardiovascular mortality. As such, the obesity status of the participants might not have been confounded by diabetes. They observed that among newly diagnosed diabetic patients, normal weight at the time of diagnosis resulted in twice the mortality rate overweight/obesity did. Arbitrarily defining normal weight/overweight/obese by discrediting BMI may lead to (1) an unrealistic step-function of risk that assumes homogeneity of risk within groups, (2) loss of power [20] inaccurate estimation, and (4) incomplete correction for confounding [21, 22]. Furthermore, it has been shown that failing to account for the negative confounding (suppression) effect of hip circumference while studying obesity-related risk might lead to biased estimates [23].

Using an inception cohort of diabetic patients, we conducted the current study aiming to reconcile the “obesity paradox” recently observed among diabetic patients. This is an attempt to examine the apparently contradictory statements regarding the obesity-mortality association and to draw conclusions to either reconcile them or explain their existence. As such, we tested the hypotheses that (1) the contributions of anthropometric measures of obesity to mortality are nonlinear and (2) the confounding bias due to hip and waist circumference might have contributed to the “obesity paradox” among diabetic patients.

## 2. Research Design and Methods

### 2.1. Study Design

Detailed descriptions of the Tehran lipid and glucose study (TLGS) have been reported elsewhere [18]; in brief, the TLGS is a large scale, long term, community-based prospective study performed on a representative sample of residents of district number 13 of Tehran, capital of Iran. Age and sex distributions of the population in the district were representative of the overall population of Tehran at the time of the baseline examination. Details of setting, measurements, and outcomes have been described in the supplementary file (see Supplementary File 1 available online at http://dx.doi.org/10.1155/2014/282089).

### 2.2. Statistical Methods

Findings on covariate variables are expressed as means (SD) and frequency (%) for continuously and categorically distributed variables, respectively. We tested for trends across BMI tertiles by using the median in each tertile as a predictor. Statistical significance of trends across BMI tertiles was examined by implementing General Linear models. The flexible parametric model for survival analysis was used to plot mortality rates (hazard function) against follow-up time. We concentrated on continuous time-dependent effect using restricted cubic spline functions to model the baseline cumulative hazard. We fitted nested models with and without spline functions of the baseline hazard and estimated the deviance (D-statistics) for each model based on the maximum likelihood *χ* ^2^. The statistical significance of the time-dependent effect was then determined by obtaining the difference in deviances of the two nested models (G-statistic) based on the maximum likelihood *χ* ^2^. Since we found no evidence of nonproportionality, the Log-Rank test and Cox test were used to examine the significance of trends in incident rates and hazard functions.

We set the statistical significance level at a two-tailed type I error of 0.05. All statistical analyses were performed using STATA version 12.0 (STATA, College Station, TX, USA).

We certify that all applicable institutional and governmental regulations concerning the ethical use of human volunteers were followed during this research. Informed written consent was obtained from all participants and the Ethical Committee of Research Institute for Endocrine Sciences approved this study. The investigations reported herein have been carried out in accordance with the principles of the Declaration of Helsinki as revised in 2000.

## 3. Results

### 3.1. Participants

Among participants aged ≥30 years, we selected those not using glucose lowering agents but diagnosed to have new onset diabetes at the baseline examination and those who developed incident diabetes during any of the two consecutive follow-up examination cycles. Complete data on covariates were available for 1,322 of participants with the median follow-up time being 9.1 years.

### 3.2. Descriptive Data

Clinical and paraclinical characteristics of participants at the time when they were first diagnosed to have diabetes are stratified by tertiles of BMI in Table 1. Age of the participants at the time they were diagnosed to have diabetes ranged from 30 to 90 years.

No consistent trend in mortality rates was observed across tertiles of BMI. In fact the first (*P* value = 0.056) and the third (*P* value = 0.588) tertiles were observed to contribute to higher mortality rates than did the middle tertile (Table 2). As shown in Table 2, adjustment for waist circumference accentuated the HRs for all-cause mortality of the first but not third BMI tertiles as compared to the middle tertile. However, further adjustment for established CVD risk factors made the statistical significance disappear.

**Figure 1:**
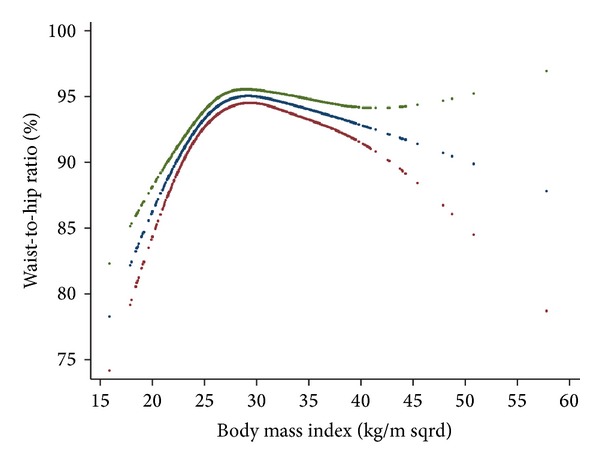
Inverse relationship of hip and waist circumference with body mass index.

As shown in Figure 1, when we plotted the waist-to-hip ratio (WHpR) against BMI, the ratio was observed to increase with increasing levels of BMI up to the threshold of ≈27 kg*·*m^−2^, where the association started to reverse. That is, as BMI increased above 27 kg*·*m^−2^, the magnitude of increases in hip circumference levels was higher than those in waist circumference.

**Figure 2:**
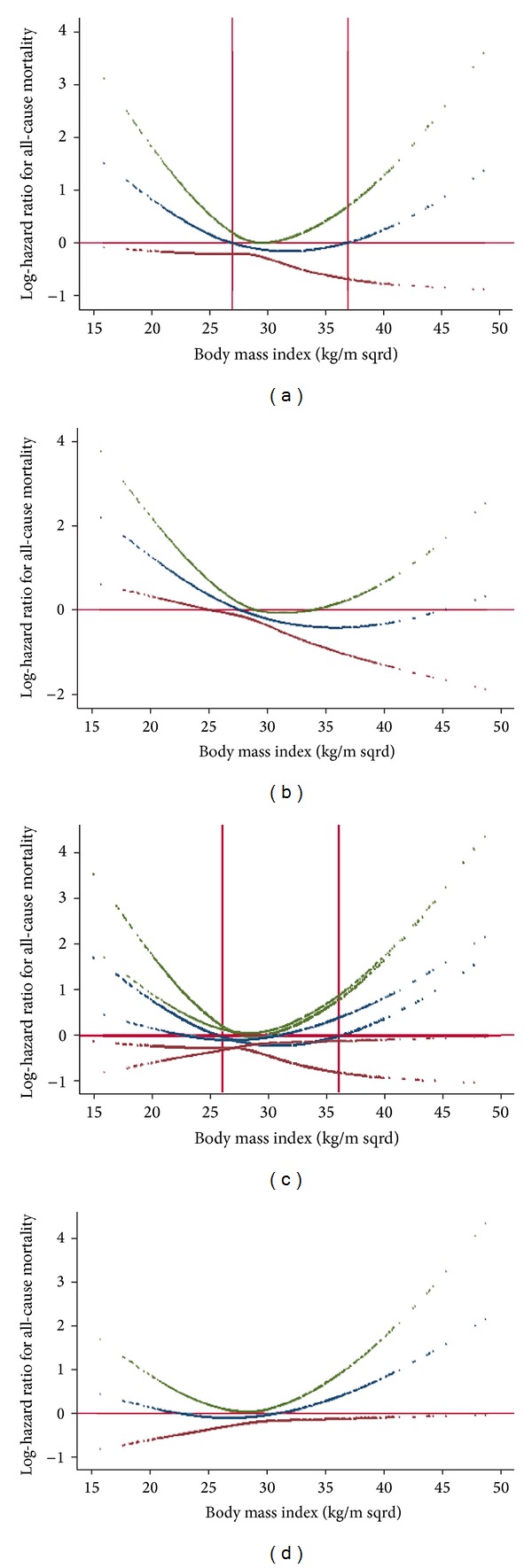
(a) Nonlinear contribution of body mass index to all-cause mortality. (b) Nonlinear contribution of body mass index to all-cause mortality, allowing for waist circumference. (c) Nonlinear contribution of body mass index to all-cause mortality, allowing for hip circumference. (d) Nonlinear contribution of body mass index to all-cause mortality, allowing for both waist and hip circumference.

Figure 2 depicts the multivariate-adjusted nonlinear contribution of the BMI to all-cause mortality adjusted for CVD risk factors only (a), CVD risk factors plus waist circumference (b), CVD risk factors plus hip circumference (c), and CVD risk factors plus both waist and hip circumference (d). As shown in the panel (a), BMI levels below 27 and above 40 kg*·*m^−2^ were associated with increased mortality. Our estimates, however, were not stable at the right-sided tail of the curve, possibly due to lack of statistical power.

As panel (b) depicts, when we added waist circumference to the BMI in the multivariate-adjusted model the steepness of BMI-mortality association curve slope for values below 27 kg*·*m^−2^ increased, whereas the steepness of BMI-mortality association curve slope for values above this threshold decreased. To examine if the increasing levels of BMI associated with decreasing trend in mortality up to BMI of 27 kg*·*m^−2^ were due to negative confounding effect of increasing hip circumference, we further adjusted the model for hip circumference and observed that the steepness of the slope of the association curve was observed to decrease considerably so that it was no longer statistically significant (panel (d)). As shown in panel (c), when we introduced hip circumference into the multivariable model already incorporating BMI, the steepness of BMI-mortality association curve slope for values below 27 kg*·*m^−2^ decreased, whereas, above this threshold, the steepness of the BMI-mortality association curve increased towards positive values.

**Figure 3:**
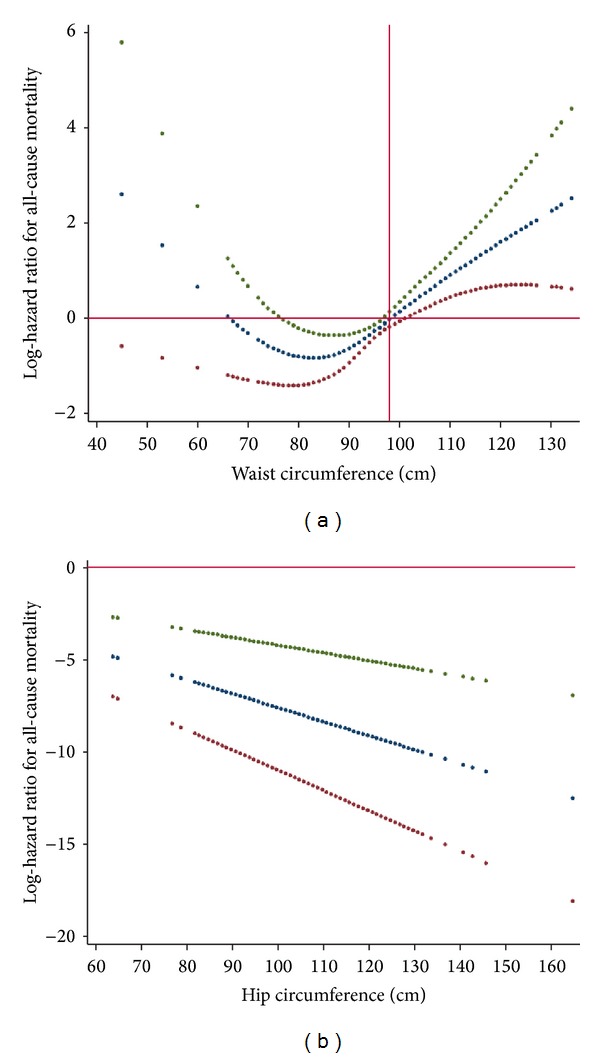
(a) Nonlinear contribution of waist circumference to all-cause mortality. (b) Linear contribution of hip circumference to all-cause mortality.

Figure 3 depicts nonlinear contribution of waist (a) and hip (b) circumference to all-cause mortality. Waist and hip circumference were mutually adjusted for each other in addition to CVD risk factors and BMI. As waist circumference exceeded the threshold of 100 cm, all-cause mortality increased in a linear fashion. As hip circumference increased, all-cause mortality decreased steadily in a linear fashion.

## 4. Discussion

### 4.1. Statement of Principal Findings

Using an inception cohort of adults with diabetes, we investigated the nonlinear contribution of the anthropometric indices of obesity to all-cause mortality allowing for potential confounding bias due to established CVD risk factors. We observed that BMI, waist, and hip circumference were all associated with all-cause mortality in a curvilinear fashion.

The finding of interest was that even across values generally considered as normal, further decreases in the BMI was associated with increased mortality. Some observations regarding this finding were as follows.

1. When the effect of waist circumference was taken into account, for values of BMI below 27 kg*·*m^−2^, the steepness of the slope increased. This implies that if waist circumference remains constant decreasing BMI would confer an excess mortality. Meanwhile, higher-than-normal values of BMI were not associated with increased mortality, except for extremely high values. That is, except for extremely high BMI values, increasing levels of BMI are safe as long as waist circumference remains constant.
2. Conversely, when we controlled our analysis for hip circumference, the opposite was true. That is, when we introduced hip circumference into the multivariable model already incorporating BMI, the steepness of BMI-mortality association curve slope for values below 27 kg*·*m^−2^ decreased, whereas, above this threshold, the steepness of the BMI-mortality association curve increased. This implies that if hip circumference remains constant, decreasing levels of BMI will not result in excess mortality. In other words, BMI-associated mortality observed in the lower extreme is merely an artifact resulting from uncontrolled confounding bias due to hip circumference.
3. An inverse U-shaped association was observed between the waist-to-hip ratio (WHpR) and BMI; that is, the ratio increased with increasing levels of BMI up to about 27 kg*·*m^−2^ and the association was reversed thereafter. In other words, the magnitude of increase in the waist circumference exceeded the increase in hip circumference up to BMI ≈ 27 kg*·*m^−2^; however, with higher BMI levels, the increase in BMI was accompanied by greater increase in hip circumference than in waist circumference. Changes in hip circumference parallels changes in waist circumference and BMI. However, in contrast to waist circumference and BMI, hip circumference inversely contributes to the mortality. As such, increased mortality due to increased waist circumference might have been counterbalanced by decreased mortality due to increases in hip circumference.
4. To conclude, If we had not considered the effect of waist circumference, we would have overestimated the mortality associated with increasing values of BMI above normal values. Also, had we not taken the effect of hip into account, we would have overestimated the mortality associated with decreasing levels on BMI below normal values.

Aforementioned observations provided evidences to support the notion that it is not obesity that is paradoxically related to all-cause mortality; rather it is BMI that harbors intermixed positive and negative confounding effects on mortality of waist and hip circumference, which have contradictory effect on all-cause mortality. To obtain an unbiased estimate of the general obesity-related health risk, in addition to waist circumference, it is crucial to control for the negative confounding effect of hip circumference. It is noteworthy that inasmuch as loss of kilograms inevitably includes loss of fat-free mass, BMI should not be ignored [24]. A better strategy, therefore, could be to use a measure of central adiposity like waist circumference and then control the association analyses for confounding bias originating from of BMI and hip circumference. Taking this approach, we observed that increases in central obesity above waist circumference of 100 cm were consistently associated with increase in all-cause mortality. Some investigators are interested in the fact that some obese individuals remain metabolically healthy and hypothesize that these individuals have a higher level of cardiovascular fitness than their metabolically abnormal obese peers and that such metabolically healthy obese individuals should demonstrate lower mortality rates [25]. In the current study, however, we adjusted our analyses for CVD risk factors so that the obesity-related mortality was independent of metabolic states. Although we have previously shown that obesity is not always associated with metabolic abnormality [26], herein, we observed that even if metabolic states remain constant increasing levels of central obesity not confounded by lean body mass or hip size were associated with increased mortality. What previous studies have referred to as the “obesity-paradox” could be reconciled in light of the fact that BMI is not a perfect measure of obesity. Although not necessarily in same direction, both body mass and fatness contribute to mortality. Such confounding bias originating from each part should be accounted for while studying obesity-related health risks, since these parameters exhibit covariation in many settings.

Some studies have suggested that waist circumference, either alone or in combination with BMI, may have a stronger relation to some health outcomes than BMI alone [27]. Waist circumference reflects abdominal or intraabdominal fat, and hip circumference reflects different aspects of body composition in the gluteofemoral region, that is, muscle, bone, and fat mass. The importance of waist and hip measurements, and the waist to hip ratio lies in the apparently different physical and metabolic characteristics of these two regions, and therefore the diverse clinical outcomes in individuals with a gynecoid (low waist to hip ratio, lower body obesity) or android (high waist to hip ratio, upper body obesity) body conformation. This may be due to the tendency for abdominal adipocytes to enlarge (hypertrophy) whereas subcutaneous femoral adipocytes increase in number (hyperplasia), perhaps due to increased levels of the adipogenic transcription factors; moreover hypertrophic adipocytes tend to be associated with dyslipidemia and insulin resistance [28].

### 4.2. Strength and Limitations

We used an inception cohort of diabetic patients which is now a standard for prognostic studies. Our patients were identified at an early and uniform point (inception) in the course of their disease. The cohort came from a large-population-based study of both sexes, with accurate and valid data on risk factors at baseline and continuous surveillance of mortality and CVD events based on standard criteria. In an attempt to reduce variability in the duration of new-onset diabetes, we restricted our analysis to participants who were not taking glucose lowering agents in the first examination. We used appropriate advanced statistical methods to capture nonlinear association of BMI, waist, and hip circumference with mortality. Both FPG and the standard 2 h-PCPG levels were available in the TLGS for identifying incident diabetes cases. Controlling the analyses for established CVD risk factors made the estimates unlikely to be affected by the bias stemming from potential confounding effects of these risk factors. Some limitations of our study merit mentioning. First, the small number of incident events precluded stratification of analyses by sex. Second, large confidence intervals on the right-sided tail of the estimated curves imply lack of statistical power and our estimates might not have been stable at high BMI, hip, or waist circumference quantities. Third, despite our best efforts to control for bias from preexisting disease, it is likely that we did not eliminate such bias completely. Thus, the increased risk of death from specific causes associated with leanness may reflect preexisting, but unrecognized, disease processes. Fourth, due to our sample, we were not able to investigate association of measures of obesity with specific causes of death. Finally, the population studied was of Persian ancestry and, thus, cannot be readily extrapolated to other populations.

### 4.3. Strengths and Weaknesses in relation to Other Studies

Despite extensive use of BMI in research and clinical practice, there are very few studies testing its diagnostic accuracy and except for that of Romero-Corral et al. no study has done this in a large, multiethnic adult population representing men and women of many age strata [29]. Even though the association between obesity and mortality is unquestionable, multiple studies worldwide have shown that overweight individuals have similar or even lower mortality when compared to people classified as having normal body weight [30, 31]. Results of these studies have challenged the association of adiposity with mortality and cardiovascular disease, when they might just represent the intrinsic limitations of BMI to differentiate adipose tissue from lean mass in intermediate BMI ranges [29].

In a pooled analysis of five longitudinal cohort studies, Carnethon et al. observed that adults who were of normal weight at the time of diagnosing diabetes had higher mortality than their overweight/obese counterparts [17]. Using arbitrary definition for normal weighted, they were not able to discover the U-shaped associations. Furthermore, failing to account for suppressive effects of increasing levels of hip circumference, which parallels the increase in BMI, might have biased their estimated mortality hazard among overweight/obese patients towards the null and thus made obesity pretend innocence while normal weight masquerading the hazard. Lean diabetic patients are recently shown to be enriched for known type 2 diabetes risk alleles compared to their obese counterparts [32]. On the other end of the spectrum, as they are already subjected to the physiological impact of obesity and insulin resistance, obese individuals presumably need fewer diabetes risk variants to become diabetic [33].

### 4.4. Implications for Clinicians

The results of studies demonstrating the obesity-paradox have consistently ignited a question of “should we start advising people to become more obese?”. We demonstrated, herein, that what previous studies have referred to as “obesity-paradox” is de facto “BMI-paradox.” Unfortunately, an implicit assumption made in many epidemiologic analyses is that BMI alone is a sufficient measure of obesity effects in regression analyses. Michels et al. have argued that this is not necessarily true and that whether BMI alone adequately captures the effect of anthropometric variables on health outcomes depends on many factors [34]. Inasmuch as BMI harbors an intermixed positive and negative confounding effect on mortality of waist and hip circumference, it cannot be directly translated to obesity. Our finding indicates that if waist circumference constantly increases, BMI does not confer any excess mortality; also, if hip circumference constantly decreases, BMI does not confer any excess mortality. It is prudent, thus, to advise people to try increasing their BMI by gaining weight via physical activities that increase muscle bulks without increasing (or even decreasing) abdominal adiposity. Clinical trials to examine the clinical usefulness of such interventions will be required in the future.

### 4.5. Unanswered Question

We have previously extensively studied different indices of obesity with respect to health risks in general population. These indices were either directly or inversely associated with mortality [35–39]. To explain excess mortality with lower BMI values, further study is required to find if the obesity-mortality association is confounded by some unmeasured risk factors that tend to accumulate as obesity decreases.

### 4.6. What Is Already Known on This Topic?

Previous studies showed that adults who were of normal weight at the time when they were first diagnosed to have diabetes had higher mortality than their overweight/obese counterparts. Using arbitrary definition for normal-weight, they were not able to discover U-shaped associations.

## 5. Conclusion

Among newly diagnosed diabetic patients, BMI, in its extremes of both leanness and obesity was associated with increased all-cause mortality, independent from established CVD risk factors. However, the association faded when we controlled our multivariate analyses for the confounding bias originating from hip and waist circumference. Our findings indicate that body mass as measured by BMI does not contribute to all-cause mortality independent from central obesity. Rather, BMI harbors intermixed positive and negative confounding effects on waist and hip circumference-related mortality. In particular, failing to control for the confounding effect of hip circumference may stymie unbiased hazard estimation. What previous studies have referred to as the “obesity-paradox” could be reconciled by the fact that BMI is not a perfect measure of obesity. Although not necessarily in the same direction, both body mass and fatness contribute to mortality. Such confounding bias originating from each part should be accounted for while studying obesity-related health risks, since these parameters exhibit covariation in many settings.

## Supplementary Material

Details of the statistical methods have been described in the supplementary file.

## Figures and Tables

**Table 1:** Baseline characteristics of the participants with new-onset diabetes mellitus stratified by tertile of body mass index.

	Tertile 1	Tertile 2	Tertile 3	*P* value∗	Total
Number of participants	441	441	440	—	1322
Median body mass index (kg*·*m^−2^)	24.9	28.9	33.8	—	28.9
Minimum body mass index (kg*·*m^−2^)	15.7	26.9	31.1	—	15.7
Maximum body mass index (kg*·*m^−2^)	26.9	31.1	57.7	—	57.7
Categorically distributed variables					
Male	259 (0.59)	213 (0.48)	118 (0.27)	<0.001	590 (0.45)
Smoker	67 (0.15)	55 (0.13)	32 (0.07)	<0.001	154 (0.12)
Blood pressure lowering drug usage	107 (0.24)	118 (0.27)	136 (0.31)	<0.001	361 (0.27)
History of previous cardiovascular disease	76 (0.18)	90 (0.21)	80 (0.18)	0.957	246 (0.19)
Assigned to life style modification intervention	160 (0.36)	170 (0.39)	166 (0.38)	0.549	496 (0.38)
Continuously distributed variables					
Age (years)	55.22 (11.96)	53.48 (11.69)	52.25 (10.93)	<0.001	53.65 (11.59)
Systolic blood pressure (mmHg)	127.40 (20.77)	130.02 (22.75)	132.52 (21.79)	<0.001	129.97 (21.86)
Total cholesterol (mmol*·*l^−1^)	5.65 (1.23)	5.84 (1.32)	5.82 (1.21)	0.001	5.77 (1.26)
High-density lipoprotein cholesterol (mmol*·*l^−1^)	1.03 (0.28)	1.01 (0.29)	1.04 (0.26)	0.002	1.03 (0.28)
Waist circumference (cm)	87.87 (8.05)	97.31 (6.98)	106.03 (8.44)	<0.001	97.06 (10.87)
Hip circumference (cm)	94.80 (5.03)	101.87 (4.56)	112.87 (5.58)	<0.001	103.16 (9.75)
Outcome					
All-cause mortality	48 (10.9)	33 (7.5)	27 (6.1)	0.236	108 (8.2)
All-cause mortality rate, per (10 000 person-year)	90.8 (65.8–125.3)	53.7 (35.0–82.3)	64.2 (44.0–93.5)	0.140	69.7 (56.3–86.2)

Data are presented as mean (SD) or frequency (%) for continuously and categorically distributed variables, respectively.

Mortality is presented as per 10 000 person-years (95% CIs).

*P values were obtained from general linear models adjusted for age for independent variables; mortality rates were compared using Cox proportional hazard regression model.

**Table 2:** Contribution of different tertiles of the body mass index to all-cause mortality.

	Hazard Ratio (95% CIs)	Std. Err.	Wald *χ* ^2^	*P* value
Model 1				
Body mass index (kg*·*m^−2^)				
First tertile	1.69 (0.99–2.88)	0.46	1.91	0.056
Second tertile	1 [reference]			
Third tertile	1.17 (0.66–2.07)	0.34	0.54	0.588
Model 2				
Body mass index (kg*·*m^−2^)				
First tertile	2.36 (1.30–4.30)	0.72	2.81	0.005
Second tertile	1 [reference]			
Third tertile	0.96 (0.52–1.77)	0.30	−0.12	0.904
Log-waist circumference [ln⁡(cm)]	20.35 (1.62–254.79)	26.24	2.34	0.019
Model 3				
Body mass index (kg*·*m^−2^)				
First tertile	1.54 (0.83–2.86)	0.49	1.38	0.168
Second tertile	1 [reference]			
Third tertile	1.49 (0.80–2.76)	0.47	1.25	0.211
Log-general cardiovascular risk	2.81 (2.11–3.75)	0.41	7.02	<0.001
Log-waist circumference [ln⁡(cm)]	0.65 (0.04–10.50)	0.93	−0.30	0.763

^†^Cox proportional hazard models were used to calculate HRs and 95% CIs. Model 1: adjusted with BMI. Model 2: adjusted with BMI and waist circumference. Model 3: adjusted with BMI, waist circumference, and general CVD risk.
