# Supplementary material for: No Obesity Paradox—BMI Incapable of Adequately Capturing the Relation of Obesity with All-Cause Mortality: An Inception Diabetes Cohort Study
Source: Int J Endocrinol. 2014 Aug 7;2014:282089. doi: 10.1155/2014/282089 (PMC4142289; doi:10.1155/2014/282089)
Supplement: Supplementary file 1 — Details of the statistical methods have been described in the supplementary file. [file 282089.f1.docx]

# Supplementary

## Study design:

Detailed descriptions of the Tehran lipid and glucose study (TLGS) have been reported elsewhere ([18](#_ENREF_18)); in brief, the TLGS is a large scale, long term, community-based prospective study performed on a representative sample of residents of district No. 13 of Tehran, capital of Iran. Age and sex distributions of the population in the district were representative of the overall population of Tehran at the time of the baseline examination.

## Setting:

The TLGS, which has two major components: a cross-sectional study of the prevalence of
non-communicable disease and associated risk factors, implemented between March 1999 and December 2001, and a prospective follow-up study. Data collection is ongoing, designed to continue for at least 20 years, with triennial examinations and annual phone calls as follow-up. A total of 27 340 residents aged ≥3 years were invited by telephone call, of which 15 010 residents participated in first examination cycle and another 3 551 residents were first examined at the second examination cycle. Participants were categorized into the cohort (n=10 394) and intervention groups (n=8 167), the latter to be educated for implementation of lifestyle modifications.

### Participants:

For the current study, among participants aged ≥30 years, we selected those not using glucose lowering agent but diagnosed with new onset diabetes at the baseline examination of the TLGS , and those who developed incident diabetes during any of the two consecutive follow-up examination cycles. A total of 1 348 participants attending the follow up study till 20th March 2010 were eligible for the current study. Complete data on covariates were available for 1 322 of participants contributing to a 12 990 person-year follow up. At the time of this study, the median follow up time was 9.1 years.

### Variables

Details on mortality and cardiovascular outcomes have been published elsewhere. (17) Parallel with triennial cycles of repeated examination in this ongoing study, every TLGS participant is first contacted by telephone. **Preliminary** information is obtained about any medical event during the previous year. They are questioned by a *trained nurse* regarding any medical conditions or whether a related event has occurred. **Complementary** data are collected by a *trained physician* during a home visit and/or a visit to the respective hospital to collect data from the participants’ medical files. In the case of mortality, data are collected from the hospital or the death certificate by an authorized local physician. Collected data are evaluated by an **outcome committee** consisting of a *principal investigator*, an *internist*, an *endocrinologist*, a *cardiologist*, an *epidemiologist*, and the *physician* who collects the outcome data. Other experts (e.g. a *neurologist*) are invited for evaluation of non-communicable disorders.

## Measurements

A trained interviewer collected information using a pretested questionnaire. The information obtained included demographic data, past medical history of cardiovascular disease (CVD), and smoking status.

Weight was measured, with subjects minimally clothed without shoes, using digital scales (Seca 707: range 0.1-150 kg) and recorded to the nearest 100 g. Height was measured in a standing position without shoes, using tape measure while shoulders were in a normal alignment. Waist circumference (WC) was measured at the umbilical level and that of the hip at the maximum level over light clothing, using an unstreched tape measure, without any pressure to body surface and measurements were recorded to the nearest 0.1 cm ([19](#_ENREF_19)). BMI (kg.m^-2^) was calculated as weight (kg) divided by square of the height (m^2^).

After a 15-minute rest in the sitting position, two measurements of blood pressure were taken, on the right arm, using a standardized mercury sphygmomanometer (calibrated by the Iranian Institute of Standards and Industrial Researches); the mean of the two measurements was considered as the participant’s blood pressure.

A blood sample was drawn between 7:00 and 9:00 AM from all study participants, after 12 to 14 hours overnight fasting. All the blood analyses were undertaken at the TLGS research laboratory on the day of blood collection. Plasma glucose was measured using an enzymatic colorimetric method with glucose oxidase. Fasting plasma glucose (FPG) measurement was performed for all participants, and the standard 2-hour post-challenge plasma glucose (2h-PCPG) test for those not on glucose-lowering drugs. Total cholesterol (TC) was assayed, using the enzymatic colorimetric method with cholesterol esterase and cholesterol oxidase. High-density lipoprotein cholesterol (HDL-C) was measured after precipitation of the apolipoprotein B containing lipoproteins with phosphotungistic acid. Analyses were performed using Pars Azmon kits (Pars Azmon Inc., Tehran, Iran) and a Selectra 2 auto-analyzer (Vital Scientific, Spankeren, Netherlands). All samples were analyzed when internal quality control met the acceptable criteria.

A previous history of CVD reflected any prior diagnosis of CVD made by a physician. Participants using oral hypoglycemic agents or insulin at the baseline examination were considered as having prevalent diabetes. To minimize the influence of diabetes duration and intentional and non-intentional weight loss secondary to the diabetes development and diagnosis, these participants were excluded from the analyses ([31](#_ENREF_31)). Otherwise, diabetes was defined either on the basis of self-reported physician-diagnosed diabetes, using glucose lowering agents, or in accordance with the American Diabetes Association (2003) definition using FPG ≥ 7.0 mmol.l^-1^ and 2h-PCPG ≥ 11.1 mmol.l^-1^ ([34](#_ENREF_34)). Current smokers were defined as participants who smoked cigarettes daily or occasionally. Using blood pressure lowering agent(s) was ascertained by self-reporting.

### Multivariate analysis

In the analysis of mortality, anthropometric measures were assessed using Cox proportional hazard regression model. To examine if the impact of anthropometric measures on mortality was modified by sex, smoking, or previous history of CVD, we introduced interaction terms for SEX× (an anthropometric measure), SMOKING×(an anthropometric measures), and history of CVD×(an anthropometric measure) into multivariate models. The significance of interaction was examined by likelihood ratio test. Neither sex, nor smoking or previous history of CVD did not significantly modify the effects of BMI on mortality (all P values for interaction >0.8). Therefore, the analyses were performed on a pooled sample to capture full sample size and statistical power. We also added Log-transformed waist and hip circumference, one at a time, to BMI in multivariate models to examine the hypothesis that obtaining waist and hip circumference measurements in addition to BMI levels helped capture the effect of BMI on mortality more precisely.

Survival time was the time from the examination date when patients were first diagnosed to have diabetes to the date of death. The censoring time of an individual was the time from the examination date when patients were first diagnosed with diabetes until the lost to follow-up date or the end of the study, whichever happened first. Censored observation meant the individuals either refused to participate further in the study (lost to follow-up), or continued until the study was ended (administrative censoring).

We controlled our regression analyses for cofounding bias due to potential confounders i.e. lifestyle modification interventions, sex, and components of the Framingham’s general CVD risk algorithm which are age, systolic blood pressure, using antihypertensive drugs, total and HDL cholesterol, diabetes, and smoking ([38](#_ENREF_38), [39](#_ENREF_39)). Using CVD as the outcome, we estimated risk of CVD for each participant. This was accomplished by incorporating the components into an accelerated failure time model. The estimated risks were then log-transformed and used as a propensity score in our final multivariate Cox proportional hazard survival regression models.

Instead of using arbitrary predetermined cut-points, we used restricted cubic splines functions of the BMI to represent its continuous relationship with mortality so that the relationships were meaningfully in accordance with substantive background knowledge. To develop the best multivariate restricted cubic splines we started with 4 knots defined at, 5th, 25th, 75th, and 95th percentiles ([40](#_ENREF_40), [41](#_ENREF_41)). In variable selection (spline selection), we dropped a variable if its removal causes a non-significant increase in deviance. We set the significance levels for covariate selection by backward elimination at 0.2. However, for BMI and hip and waist circumference, we first set the significance level at unity, forcing them into the model, leaving others to be selected or not. We were then guided by the shapes of curves obtained as well as literature as to how to select models.

To examine the simultaneous changes in waist and hip circumference across different levels of BMI, we used waist-to-hip ratio (WHpR) to present the simultaneous changes in waist and hip circumferences with varying levels of BMI.

As a sensitivity analysis we excluded physician-diagnosed patients at the baseline examination and repeated all analysis. The shape and strength of association remained essentially unchanged. Thus, results from whole sample were reported.
